# *Pneumocystis jiroveci* Pneumonia: A Review of Management in Human Immunodeficiency Virus (HIV) and Non-HIV Immunocompromised Patients

**DOI:** 10.1055/s-0043-1764375

**Published:** 2023-03-24

**Authors:** Atif Ibrahim, Asmi Chattaraj, Qamar Iqbal, Ali Anjum, Mohammad Ebad Ur Rehman, Zobia Aijaz, Fazila Nasir, Sadia Ansar, Tirdad T. Zangeneh, Ahmad Iftikhar

**Affiliations:** 1 North Mississippi Medical Center, Tupelo, Mississippi, United States; 2 University of Pittsburgh Medical Center, McKeesport, Pennsylvania, United States; 3 TidalHealth, Salisbury, Maryland, United States; 4 King Edward Medical University, Lahore, Pakistan; 5 Rawalpindi Medical University, Rawalpindi, Pakistan; 6 Dow Medical College, Karachi, Pakistan; 7 Allama Iqbal Medical College, Lahore, Pakistan; 8 Rawal Institute of Health Sciences, Islamabad, Pakistan; 9 Division of Infectious Diseases, Department of Medicine, University of Arizona, Tucson, Arizona, United States; 10 Department of Internal Medicine, University of Arizona, Tucson, Arizona, United States

**Keywords:** *pneumocystis jirovecii* pneumonia, *pneumocystis carinii* pneumonia, PJP in HIV, PJP treatment, PJP prophylaxis, steroids in PJP

## Abstract

*Pneumocystis jirovecii* pneumonia is an opportunistic fungal infection that was mainly associated with pneumonia in patients with advanced human immunodeficiency virus (HIV) disease. There has been a decline in *Pneumocystis jirovecii* pneumonia incidence in HIV since the introduction of antiretroviral medications. However, its incidence is increasing in non-HIV immunocompromised patients including those with solid organ transplantation, hematopoietic stem cell transplantation, solid organ tumors, autoimmune deficiencies, and primary immunodeficiency disorders. We aim to review and summarize the etiology, epidemiology, clinical presentation, diagnosis, and management of *Pneumocystis jirovecii* pneumonia in HIV, and non-HIV patients. HIV patients usually have mild-to-severe symptoms, while non-HIV patients present with a rapidly progressing disease. Induced sputum or bronchoalveolar lavage fluid can be used to make a definitive diagnosis of *Pneumocystis jirovecii* pneumonia. Trimethoprim-sulfamethoxazole is considered to be the first-line drug for treatment and has proven to be highly effective for *Pneumocystis jirovecii* pneumonia prophylaxis in both HIV and non-HIV patients. Pentamidine, atovaquone, clindamycin, and primaquine are used as second-line agents. While several diagnostic tests, treatments, and prophylactic regimes are available at our disposal, there is need for more research to prevent and manage this disease more effectively.

## Introduction

*Pneumocystis jirovecii* pneumonia (PJP) or *Pneumocystis* pneumonia formerly known as *Pneumocystis carinii* pneumonia is an opportunistic fungal infection that was mainly associated with pneumonia in patients with advanced human immunodeficiency virus (HIV) disease. However, its incidence has been on the decline ever since the implementation of antiretroviral therapies (ART) in the treatment of HIV disease. PJP also affects non-HIV immunocompromised patients such as those after solid organ transplantation (SOT) and hematopoietic stem cell transplantation (HSCT), malignancy, and chronic inflammatory conditions treated with immunosuppressive medications including high dose steroids. The incidence trend in the latter group is consistently on the rise.

In the following review, we present a comprehensive review of PJP infection, describing the history, nomenclature, epidemiology, transmission, clinical manifestations, management, and the use of prophylaxis in at-risk adult population.

## Methodology

This review was conducted by researching relevant articles using PubMed, Cochrane, and Google Scholar databases using various combinations of keywords such as, PJP, *Pneumocystis carinii* , *Pneumocystis carinii* pneumonia, PJP, immunocompromised, treatment, prophylaxis, HIV, non-HIV, epidemiology, symptomatology, risk factors, diagnosis, SOT, malignancy, autoimmune disorders, pregnancy, prophylaxis, and prognosis.

## Etiology

The *Pneumocystis carinii* organism was first identified by Chagas in 1909, who mistook it for *Trypanosoma cruzi* . Initially considered to be a protozoan, *P. carinii* was recategorized as a fungus, based on genomic evidence. 1 Variations were also found between Pneumocystis organisms isolated from different host species, showing that PJP is not a zoonotic disease. This led to nomenclature changes, with the organism responsible for causing disease in humans being renamed *Pneumocystis jirovecii* .

*Pneumocystis jirovecii* is a unicellular, obligate fungus that has two morphological forms: the cyst, a thick-walled spherical or ovoid structure; and the trophozoite, a thin-walled excysted sporozoite. *Pneumocystis* was initially misclassified as a protozoan because of its atypical characteristics. It lacks chitin in its cell wall and its cell membrane contains cholesterol instead of ergosterol, making it resistant to antifungal drugs that target ergosterol, such as azoles and amphotericin B. 2

## Epidemiology

PJP has been known to cause disease mostly in immunocompromised individuals. The first clinical case of *pneumocystis* was reported during World War 2 among premature and malnourished children. In 1981, *pneumocystis* was initially reported among persons who inject drugs and men who have sex with men with HIV/AIDS. 3

*Pneumocystis jirovecii* can colonize the lungs without causing pneumonia that may be associated with increased risk of transmission. Prophylaxis use in colonized individuals may lead to drug-resistant mutations. Moreover, low level of *Pneumocystis jirovecii* may stimulate inflammatory response in lungs that can lead to chronic obstructive pulmonary disease. 4 Asymptomatic or subclinical infection of infants is common. 5 Studies of *Pneumocystis jirovecii* colonization among healthy adults in published studies are fraught with contradictions, but it clearly occurs in HIV infected and other immunosuppressed individuals.

Various studies have described the incidence of PJP with different underlying conditions some of which are shown in Table 1 . In developed countries, the use of prophylactic drugs has decreased PJP infections in HIV patients, but it is still increasing in non-HIV patients treated with immunosuppressive medications and chemotherapy.

**Table 1:** Incidence of PJP with different underlying conditions

Author(s)	Year	Method and duration of study	Number of PJP cases	Risk factors
Gaborit et al 87	2019	Prospective cohort study (Jan 2012–Jan 2017)	107	• HIV positive ( *n* = 21, 20%)
				• Hematological malignancy ( *n* = 37, 35%)
				• Solid organ transplant ( *n* = 27, 25%)
				• Systemic disease ( *n* = 13, 12%)
				• Solid tumors ( *n* = 12, 11%)
				• Primary immune deficiency ( *n* = 6, 8%)
Pereira-D **í** az et al 96	2019	Observational, descriptive transversal study (2008–2012)	4,554	• HIV positive ( *n* = 3350, 73.5%)
				• HIV negative ( *n* = 1204, 26.4%) Hematological malignancies ( *n* = 349, 29%) Chronic lung diseases ( *n* = 192, 15.9%) Other malignancies ( *n* = 179, 14.9%) Autoimmune diseases ( *n* = 93, 7.7%) Chronic nephropathies ( *n* = 68, 5.6%) Chemotherapy ( *n* = 59, 5%) Transplant ( *n* = 54, 4.5%) Hematological disorders ( *n* = 21, 1.7%) Chronic liver disease ( *n* = 14, 1.2%) Unknown (176, 14.5%)
Ricciardi et al 97	2017	Retrospective cohort study (Jan 2011–Jan 2015)	116	• HIV positive (22.4%)
				• Hematological and HSCT (37.9%)
				• Chronic lung disease (16.4%)
				• Solid cancer (7.8%)
				• Solid organ transplant (3.4%)
				• Others (12%)
Roux et al 98	2014	Prospective cohort study (Jan 2007–Dec 2010)	544	• HIV positive ( *n* = 223, 41%)
				• HIV negative ( *n* = 321, 59%) Solid organ transplant ( *n* = 99, 30.8%) Hematological malignancy ( *n* = 84, 26.2%) HSCT ( *n* = 27, 8.4%) Autoimmune ( *n* = 65, 20.5%) Solid organ malignancy ( *n* = 46, 14.3%)

Abbreviations: HSCT, hematopoietic stem cell transplantation; HIV, human immunodeficiency virus; PJP, *Pneumocystis jirovecii* pneumonia.

## Pathophysiology

PJP organisms enter the alveoli through the respiratory passages. They adhere to type 1 pneumocytes, causing diffuse alveolar damage by eroding the pneumocytes. 6 Normally, the host immune response eradicates the fungus, particularly by the action of alveolar macrophages. In immunocompromised patients; however, a decline in CD4 T-lymphocytes is associated with a CD8 T cell mediated immune response, which causes tissue damage and impairs gaseous exchange without eliminating the fungus. 7 Inhibition of phospholipid secretion by a surface antigen, known as MSG antigen, has also been implicated in pathogenesis of PJP, producing surfactant changes and leading to impaired gas exchange. 8 Histological examination of lung tissue infected by *Pneumocystis jirovecii* shows the presence of an eosinophilic amorphous exudates in the alveolar spaces, giving the tissue a honeycomb appearance. Lymphocytic infiltration of the interstitium is also seen.

## Clinical Presentation and Risk Factors

The signs and symptoms of PJP infection are non-specific. Due to the overlapping nature of symptoms, obtaining a thorough history plays a pivotal role in establishing the diagnosis promptly. A relevant history of HIV disease, malignancy, high dose steroid use, and/or immunosuppressive drugs utilization is important. 9 The triad of PJP symptoms (dry cough 95%, progressive dyspnea 95%, and low-grade fever 80%) is well established in HIV patients. 10 Similar symptoms could present with severe and rapid onset (5–6 days compared with 25–28 days in HIV patients) in non-HIV patients. 11 PJP can also present as pneumothorax with chest pain and shortness of breath in 2 to 4% of the patients. 12 Severe PJP disease causes significant hypoxia with a higher alveolar-arterial (A-a) gradient and respiratory failure with overall poor outcome. Non-HIV patients may present with a rapidly progressing disease likely due to an intense immune response to PJP in the lungs than in HIV patients. 9 Mortality in severe cases in non-HIV immunocompromised patients is 30 to 60% versus 10 to 20% in HIV patients. 13

Multiple risk factors are known to increase the risk of PJP infection. Since its discovery, PJP is believed to be the disease of the immunocompromised. In HIV patients, CD4 counts of less than 200, high HIV viral load, recurrent bacterial pneumonia, prior history of PJP infection, and weight loss are considered as risk factors. 14 Cellular immunity defects, severe combined immunodeficiency, X-linked hyper-immunoglobulin M syndrome, Good's syndrome, hematological malignancies (CLL), hematopoietic stem cell transplant (HSCT), SOT, rheumatological disease (polyarteritis nodosa, granulomatosis with polyangiitis, dermatomyositis/polymyositis, ulcerative colitis, rheumatoid interstitial lung diseases), steroids use, utilization of Rituximab, Alemtuzumab, and tumor necrosis factor inhibitors are also known risk factors. 15 16 17 18 19

## Differential Diagnosis

The differential diagnosis of PJP includes acute respiratory distress syndrome, tuberculosis, cytomegalovirus (CMV), Legionella pneumonia, mycoplasma pneumonia, other viral or bacterial pneumonias, pulmonary embolism, lymphocytic interstitial pneumonia, and coronavirus disease 2019infection. 20

## Diagnosis

Early diagnosis of PJP is an absolute imperative when it comes to its management and disease outcomes. For this purpose, various imaging and laboratory tests are used to establish diagnosis.

Characteristic findings of PJP on a chest radiograph include bilateral interstitial pattern characterized by finely granular, reticular or ground-glass opacities. When the chest radiograph is inconclusive, but the history and clinical presentation are suggestive of PJP, chest computed tomography (CT) imaging is recommended as it has a higher sensitivity for diagnosing PJP. Ground-glass attenuation is a characteristic finding on chest CT. Even though the absence of ground-glass opacities on CT chest can exclude the PJP infection, there are case reports showing PJP presenting with diffuse nodular patterns, unilateral infiltrates and, rarely, as solitary pulmonary nodules. 21 22 While non-HIV immunocompromised patients mostly present with typical ground-glass opacities, cystic pulmonary lesions can also be seen in HIV immunocompromised patients. 23

Cultures are not commonly used in the diagnosis of PJP due to limitations in growth of *Pneumocystis jirovecii* through these techniques. Therefore, definitive diagnosis of PJP requires application of molecular and genetic or direct visualization techniques on induced sputum or bronchoalveolar lavage (BAL) fluid for the detection of *Pneumocystis jirovecii* . Induced sputum may be initially used first for microscopy with staining or molecular and other testing and if not diagnostic then BAL fluid may be utilized, although more invasive but with a better diagnostic yield.

Nested polymerase chain reaction (PCR) has a sensitivity and specificity of close to 100 percent in detecting PJP in HIV/AIDS patients; while in non-HIV immunocompromised patients, it may not be an ideal tool for diagnosis. This has been illustrated in studies that sensitivity and specificity of nested PCR reduce to 25 and 44%, respectively, in immunocompromised patients with lymphoma and leukemia. 24 Azoulay et al showed PCR sensitivity of 87.2% and specificity of 92.2% with positive and negative predictive values of 51.5 and 98.7%, respectively. 25 However, it cannot always distinguish infection from colonization. Hence, in clinical practice, physicians are always encouraged to correlate respiratory symptoms, radiological and laboratory findings for diagnosing suspected cases of PJP.

Clinical specimens can also be subjected to immunofluorescence and standard staining techniques (Grocott methenamine silver stain) for detecting *Pneumocystis jirovecii* . Both direct and indirect fluorescent antibody tests can be used for the detecting *Pneumocystis jirovecii* in induced sputum or BAL fluid.

Serum 1–3 β D Glucan testing can be used for diagnosing PJP and can be a good diagnostic test for patients who cannot undergo invasive procedures for specimen collection required for a definitive diagnosis. 26 Serum 1–3 β-glucan levels have higher false-positive results especially in certain conditions such as use of cellulose membranes for hemodialysis, application of glucan-containing gauzes, administration of albumin, intravenous (IV) immunoglobulin and blood products produced, presence of gram-negative endotoxemia, and use of certain antimicrobials, such as cephalosporin and β-lactam–β-lactamase inhibitor combinations. Moreover, elevated serum 1–3 β-glucan can also be present in various fungal infections such as invasive aspergillosis and systemic candidiasis further decreasing its specificity. 27 Metagenomic next-generation sequencing is relatively a newer modality for diagnosing PJP and has shown promising results. 28

## Management

Management of PJP is a well-researched topic. Many clinical trials, retrospective, or case–control studies have been done since the 1990s to look further into different drug regimens suited for treating PJP. PJP can best be managed by an interdisciplinary team consisting of pulmonologists, infectious diseases experts, pharmacists, intensive care specialists, nurses, and respiratory therapists. The first-line therapy for PJP is trimethoprim-sulfamethoxazole (TMP/SMX), regardless of the underlying comorbidity. Alternative regimens available include pentamidine, atovaquone, clindamycin, and primaquine and vary according to response to the first-line agent, and the severity of disease, which also determine the use of corticosteroids. Since *Pneumocystis jirovecii* does not contain ergosterol in the cell wall, polyenes or azoles antifungals are not effective. The following underlying conditions are associated with PJP and on whom most of the research has been focused.

## HIV Infection

First-line and alternative therapies available are outlined in Table 2 . 29 30 31 The duration of therapy is usually 21 days, though longer courses may be required depending on the response and severity. 13

**Table 2:** Summary of the first- and second-line treatment for PJP in different disease population

	HIV 29	Solid organ transplant 30	Malignancy 31
**Treatment**	PJP (moderate-to-severe) First-line treatment: • TMP-SMX: IV TMP 15–20 mg/kg/day + SMX 75–100 mg/kg/day given every 6–8 hour, can convert to PO after improvement. *Alternative:* • IV pentamidine 4 mg/kg OD; dose reduction to IV pentamidine 3 mg/kg OD in case toxicity develops *or* • PO primaquine 30 mg OD + Clindamycin:IV 600 mg q6h or 900 mg q8h or PO 450 mg q6h or 600 mg q8h. PJP (mild-to-moderate) *First-line treatment:* • TMP-SMX: PO TMP 15–20 mg/kg/day + PO SMX 75–100 mg/kg/day, *or* • TMP-SMX double strength—2 tabs PO TID *Alternative:* • PO dapsone 100 mg OD + PO TMP 15 mg/kg/day or • PO primaquine 30 mg OD + clindamycin PO - 450 mg q6h or 600 mg q8h, *or* • PO atovaquone 750 mg BID **Adjunctive corticosteroids** PJP (moderate-to-severe) infection meeting criteria: • PaO2 <70 mm Hg in RA *or* • Aa DO2 gradient ≥35 mm Hg Regimen: • Prednisone (within 72 hours of PJP treatment or as soon as possible) • Day 1 to 5: PO 40 mg BID • Day 6 to 10: PO 40 mg OD • Day 11 to 21: PO 20 mg OD • IV methylprednisolone can also be used, alternatively	( *Preferred* ) TMP-SMX: IV TMP 15–20 mg/kg/day + SMX 75–100 mg/kg/day or P.O. (divided in TID/ QID dosing) *(alternative therapies)* *Pentamidine* **:** IV 4 mg/kg/day. OR *Atovaquone* **:** PO 750 mg BID/ TID OR *Clindamycin and primaquine* **:** PO primaquine 15–30 mg OD + IV clindamycin 600mg QID/ PO 350–400 mg TID/QID *OR* PO dapsone 100 mg OD (single agent) or combined with PO TMP 15 mg/kg/day TID.	First line **:** • TMP-SMX:15–20 mg/kg of TMPalong with 75–100 mg/kg of SMX per day for ≥14 days ( *preferred* ) OR • IV pentamidine: 4 mg/kg/day OR • Primaquine + clindamycin:PO 30 mg/day + IV/PO 600 mg TID OR • Atovaquone:PO 750 mg BID/TID Second-line options (salvage): • Primaquine 30 mg daily + clindamycin 600 mg TID OR • IV pentamidine 4 mg/kg/day OR • TMP/SMX 15–20 mg/kg/day +caspofungin 70–50 mg/day

Abbreviations: Aa, alveolar–arterial; BID, twice daily; IV, intravenous; LD, loading dose; OD, once daily; PJP, *Pneumocystis jirovecii* pneumonia; PO, per oral; q6h, every 6 hours; q8h, every 8 hours; RA, room air; TID, three time a day; TMP-SMX, trimethoprim-sulfamethoxazole.

Corticosteroids have shown to be efficacious in HIV-infected individuals with moderate-to-severe disease. A meta-analysis by Wang et al, which included 548 cases of HIV—PJP across six randomized controlled trials (RCTs) used adjunctive corticosteroids along with TMP-SMX or pentamidine for the treatment of PJP in HIV patients. The risk of mortality in the experimental group was half as compared with the control group, and hence showing that use of adjunctive corticosteroids may reduce mortality. 32 In the three-center study of 82 patients by Benfield et al, treatment response rates were almost comparable between TMP-SMX (73%) and clindamycin-primaquine (68%). The response rate was significantly lower with pentamidine with increased mortality at 3 months and atovaquone showed no significant benefit as a second-line treatment. 33 In a multicenter, retrospective cohort study at nine hospitals in Taiwan, the clinical effectiveness and safety of echinocandins were evaluated over 5 years. The in-hospital mortality rate was similar between patients receiving echinocandins only vs those receiving echinocandins and TMP-SMX, although this is a small study and has not impacted clinical practice. 34

## Solid Organ Transplantation

SOT patients are particularly at risk of PJP due to immunosuppressive therapies. Also, acute T cell-mediated rejection (requiring prolonged and stronger immunosuppression beyond steroids) and treatment with rituximab significantly increase the risk of PJP. 35 36 37 One retrospective study analyzed the correlation of serum biomarkers in the clinical course of PJP, and found that serum surfactant protein-D values increase when there is failure in non-HIV PJP, but no such correlation was found with β-D glucan, Krebs von den Lungen-6 antigen levels. 38

A study comparing low-dose TMP (<15 mg/kg/day) with the standard dose of TMP (15–20 mg/kg) found no difference in the mortality rates, but significantly reduced adverse effects. 39 IV pentamidine is avoided in pancreas and islet cell transplants to avoid potential islet cell necrosis. 30 40

The duration of treatment is a at least 14 days in mild-to-moderate and 21 days for severe disease. In SOT patients, corticosteroid use is controversial. Cases that are moderate-to-severe in intensity, prednisone 40 to 60 mg given twice daily for a total of 5 to 7 days started within 72 hours of diagnosis is recommended, reducing dose slowly over the next 7 to 14 days. 30 One retrospective study compared mortality rates between high-dose, low-dose, and no steroids in PJP-affected patients with malignancies and organ transplant and found no mortality benefits. 41 A retrospective cohort study of 323 HIV-negative PJP patients at the Mayo Clinic over 10 years showed that the use of steroids within 72 hours did not have any impact on intensive care unit (ICU) admission or length of stay, mortality, or intubation rates. 42

## Hematological Malignancies and Solid Tumors

Recommendations for the treatment of PJP in hematology patients have been provided by European Conference on Infections Leukemia as detailed in Table 2 .

The PJP severity grading system of Miller et al 43 is used in non-HIV patients as the usual CURB-65, pneumonia severity index underestimates the severity. 31 This grading not only uses oxygen saturation values, but also other parameters like respiratory rate, age, comorbidities, or additional organ dysfunction. In the absence of improvement after 8 days of treatment, this could likely be treatment failure and consideration should be given to repeat investigations including CT chest and bronchoscopy to identify coinfections. 29 41 However, persistent *Pneumocystis jirovecii* PCR positivity in repeat BAL sample is not suggestive of treatment failure. 29 31 44 Mechanical ventilation may be required for patients presenting with acute hypoxic respiratory failure, which is not uncommon with underlying malignancies.

As with SOT recipients, adjunctive corticosteroids are controversial, due to the varying results of retrospective observational studies and scarcity of RCTs in HIV-negative patients. A meta-analysis showed that although individual studies reported shorter intensive care unit stay and duration of mechanical ventilation with adjunctive corticosteroids, there was no statistically or clinically significant association between corticosteroids and survival in non-HIV-PJP patients. Fishman and Gans 30 and McKinnell et al 45 also suggested using corticosteroids in severe non-HIV PJP based on their findings of shorter ICU stay and shorter clinical course.

## Autoimmune Disorders

The use of immunosuppressive drugs in the treatment of rheumatological disorders is on rise with an increase in the incidence of PJP infection. Unlike other non-HIV patients, factors establishing the severity and mortality of PJP infection in this group are not well known. It was noticed that high-dose steroids (>40mg/day) along with cyclophosphamide use in systemic lupus erythematosus and dermatomyositis/myositis patients had led to increased PJP infections. Need for mechanical ventilation, hemodynamic instability, and low oxygenation were found to be poor prognostic factors. 46 Rheumatoid arthritis patients who had developed PJP infection while taking infliximab were found to have lower serum albumin, lower serum immunoglobulin G levels, and had been treated with steroids. 47 Patients with glomerulonephritis, treated with immunosuppressive therapy who developed PJP, tended to have higher blood urea nitrogen (BUN), higher serum creatinine, and lower hemoglobin levels. 48 About 29% mortality was reported in giant cell arteritis patients taking high-dose steroids (50mg/day) who developed PJP infection treated with TMP/SMX as first-line therapy. 49

## Primary Immunodeficiency Disorders

Primary immune deficiencies such as hyper-immunoglobulin E (IgE) syndrome are known to be associated with PJP along with other spectra of infections. The response of TMP/SMX treatment on PJP infection in hyper-IgE syndrome was found to be good with four out of five patients living after treatment with TMP/SMX with follow-up ranging from 4 months to 15 years. 50

## Pregnancy

Pregnant patients are at mildly increased risk of PJP infection due to cellular immunity modifications during pregnancy. 51 It was found that up to 15.5% of pregnant patients are asymptomatic colonizers. 52 In a case series of HIV pregnant patients, 60% of the patients treated with TMP/SMX as first line or IV pentamidine as second line were able to be discharged from the hospital. 53

## Pediatric Patients

There is not much difference in the clinical manifestations, complications, and mortality between pediatric and adult populations. PJP infection tends to occur more commonly in the setting of malignancy and transplant versus malignancy and HIV in adults. 54 PJP infection is not seen frequently in children who are treated with chronic steroid therapy and when prophylaxis is used but hypersensitivity reactions and bone marrow suppression are commonly encountered. 55 Radiological findings in children are similar to adults with the CT scan remaining as the imaging modality of choice. 56 Other diagnostic utilities are the same as in adults with BAL the preferred method. Limited data are available on other molecular and genetic tests in children. 57 While in adults, use of alternative anti-PJP drugs is common, children have less reports of receiving alternative medications for treatment. 54 Similarly, CMV infection incidence is also less common in children. 54 According to the Centers for Disease Control and Prevention recommendations, HIV-infected infants/children aged between 1 to 5 and 6 to 12 years with CD counts less than 500 cells/mm3 and less than 200 cells/mm3 should receive prophylaxis in the form of TMP/SMX 150/750 mg/m ^2^ BSA per day into two divided doses, while dapsone and atovaquone are the alternatives. The treatment of choice is TMP/SMX dosed as TMP 15 to 20 mg/kg per body weight, SMX 75 to 100 mg/kg per body weight IV, or per oral in 3-4 divided doses. Alternative therapies consist of Pentamidine and atovaquone. 57 Caspofungin in combination with TMP/SMX has shown good results in pediatric immunocompromised patients. 58 The criteria for steroids use are similar to adults. Dapsone and primaquine, though used in adults, have limited data available in pediatric populations. 57 In pediatric patients infected with HIV, factors such as age less than 6 months, respiratory rate more than 59/min, O2 saturations less than or equal to 92%, and the absence of vomiting are independently associated with the diagnosis of PJP and warrant empirical treatment with antipneumocytosis antibiotics. 59

## Treatment Resistance

Antimicrobial resistance during treatment has been reported in PJP infections. A potential reason for resistance is thought to be the polymorphism in genes targeting anti-PJP therapies. Mutation at dihydrofolate reductase and dihydropteroate synthase (DHPS) can cause resistance to TMP/SMX treatments. 60 However, a 144V mutation in cytochrome b can cause resistance to atovaquone. Prior exposure to sulfa and sulfonamides and prior history of PJP infection increase the risk of having mutations in the DHPS genotype. 61 DHPS mutation was found in 48% of the HIV patients with PJP infection. It was noted that patients who had DHPS mutation had longer stay on mechanical ventilators. It was also found that patients with mutations had longer lengths of stay in hospital. 62

## Prophylaxis

Prophylaxis for PJP is recommended in patients with impaired immunity. Decision to start prophylaxis depends on the risk stratification of patients. According to a study, PJP prophylaxis should be started in adults with greater than 3.5% risk of contracting PJP. 63

It is recommended that chemoprophylaxis be started in HIV patients with CD4 cell count less than 200/mm ^3^ . Prophylaxis against PJP in autoimmune and/or inflammatory diseases is recommended in case of absolute peripheral lymphopenia, high doses of corticosteroids, combination with other immunosuppressive agents, and concomitant lung diseases. 64

Patients with lymphoma that were treated with rituximab containing regimens frequently developed PJP and prophylaxis in these patients have proven to be very beneficial. 65 Similarly, PJP has been reported in solid tumor malignancies. According to a study in lung cancer patients, radiotherapy, concurrent chemo radiotherapy, lymphopenia, and high-dose steroid (20mg prednisolone equivalent for greater than or equal to 3 weeks) were associated with PJP development prompting PJP prophylaxis initiation. 66 In patients receiving SOT, chances for developing PJP are highest during the first 6 months in cases with following risk factors: low lymphocyte counts, CMV infection, hypogammaglobulinemia, treated graft rejection or corticosteroids, and advancing patient age (>65). Therefore, PJP prophylaxis with TMP-SMX is recommended. 30

TMP-SMX is considered to be first-line drug and has proven to be highly effective for PJP prophylaxis in both HIV and non-HIV patients. 67 68 Dapsone can also be used for primary prophylaxis of PJP, but its safety for secondary prophylaxis has not been established. 69 Aerosolized pentamidine has a better safety profile than TMP-SMX but has not been as successful in preventing PJP. 70 Atovaquone can also be considered for PJP prophylaxis in patients that cannot tolerate TMP-SMX. 71 It is worth mentioning that breakthrough infections are reported in patients taking atovaquone for the treatment or prevention of PJP infection. 72

Many outbreaks of PJP have been reported particularly in transplant patients. To prevent these outbreaks, PJP prophylaxis is recommended in transplant patients that are exposed to PJP cases ( Table 3 ). 73 74 75 76 77 78

**Table 3:** Chemoprophylaxis regimens for *Pneumocystis jirovecii* pneumonia

Chemoprophylaxis for *Pneumocystis* pneumonia 73			
Drugs	Mechanism of action	Dosage	
		Adults	Children
Trimethoprim-sulfamethoxazole	These two drugs act synergistically to inhibit folic acid synthesis 74 75	80 mg/400 mg once daily or 160 mg/800 mg once daily or 160 mg/800 mg three times a week	In children older than 1 month, 75 mg/m ^2^ /dose or 2.5 mg/kg/dose (maximum 160 mg/dose) orally, Q8H, three times per week
Dapsone	Inhibits dihydrofolic acid synthesis in *Pneumocystis jirovecci* 76	100 mg daily	In children older than 1 month, 2 mg/kg/dose once per day or 4 mg/kg/dose once per week
Pentamidine	Inhibits glucose metabolism, protein synthesis and amino acid transport in *Pneumocystis jirovecci* 77	300 mg inhaled once every 28 days	In children older than 2 years, 4 mg/kg/dose (maximum dose 300 mg) IV every 28 days. In children older than 5 years, 300 mg inhaled once every 28 days
Atovaquone	Inhibits ubiquinone binding to *cytochrome b* in *Pneumocystis jirovecci* mitochondria 78	1,500 mg once per day	In children between age of 1–3 months, 30 mg/kg/dose once every day In children 4–24 months old, 45 mg/kg/dose (maximum 1,500 mg) once per day In children older than 24 months, 30 mg/kg/dose (maximum 1500 mg) once per day

Abbreviation: IV, intravenous.

## Outcomes and Prognosis

With the advent of effective antiviral therapy, the number of PJP cases have decreased in HIV patients. Early initiation of PJP treatment improves outcomes, while some patients continue to develop severe disease and respiratory failure. Mortality is high in patients who need intensive care or mechanical ventilation. The degree of hypoxemia (A-a Gradient) correlates with response to treatment and outcomes of PJP infection in HIV patients. The adjunctive corticosteroids have proven improved clinical outcomes in HIV patients. 79 Other factors that are associated with poor outcomes include age, history of previous PJP infection, an elevated lactate dehydrogenase (LDH) level, a low CD4 cell count, and coinfection with CMV. 80 81 Outcomes are poor in patients who develop pneumothorax spontaneously or with use of mechanical ventilation. 82 The genotyping profile of PJP has shown that DHPS mutations is associated with worse outcomes and development of severe PJP infection. 83

In comparison to HIV patients, PJP infection in non-HIV patients is associated with a high mortality and morbidity even after treatment initiation (10–20% vs. 30–60%). 84 Many studies have reported prognostic factors, but due to heterogeneity in patient's baseline, the data results are divergent. In a meta-analysis conducted by Liu et al, the mortality in non-HIV patients depends on the underlying risk. Among them, patients with malignancy have higher risk than transplants recipients or populations with connective tissue disorders. Factors such as age, body mass index, coinfections, mechanical ventilation, development of pneumothorax, LDH levels, and lymphocyte count are all related to the prognosis of PJP infections. 85

Low peripheral lymphocyte subset counts have unfavorable outcomes in non-HIV patients. CD8+ T cell count less than 300/μL is considered an independent predictor for mortality. 86 The presence of alveolitis (>10% lymphocyte and 5% neutrophils in BAL fluid cytology) is a favorable predictor of 90-day mortality and suggestive of less severe infection. 87 Other laboratory parameters such as LDH levels are a useful predictor for survival and can aid clinicians in early recognition of severe PJP infection, but specific values are not determined. Beta-glucan level is a reliable marker for PJP infection and can be performed before BAL, but the prognostic value is low and not specific for PJP infections. 88

The high rates of hospitalization and intensive care unit admission are common in non-HIV PJP patients. Also, noninvasive mechanical ventilation fails in a higher proportion of HIV-negative cases compared with HIV-positive cases. 89 Prediction scores used in ICU such as A-Drop, as well as BUN/albumin ratio help assess 30-day mortality. 90 Festic et al described APACHE III score, intubation delay, longer duration of positive predictive value, and pneumothorax as unfavorable outcomes in patients admitted to ICU. 91 The radiologic evidence of crazy paving ground-glass opacities on high-resolution CT has also been associated with a poor prognosis. 92 Among non-HIV oncological patients, the risk of PJP is increased in hematological malignancy but patients with solid tumors have a much worse prognosis and increased mortality. Coinfection with *aspergillu* s species is common in immunocompromised patients and associated with severe disease in such patients. 85 The role of early adjunctive glucocorticoids in non-HIV hematology and oncology patients has not shown a great benefit in decreasing 30-day and 1-year mortality rates. With regard to intubation and mechanical ventilation, risk reduction with the use of adjunctive glucocorticoids is still inconclusive. 93 Older age and coexisting interstitial lung disease are poor prognostic factors in patients with connective tissue disorder. The treatment is standard TMP-SMX, but atovaquone has shown better outcomes than pentamidine. 94

A risk nomogram (graphical models that enable users to calculate the overall probability of a specific outcome for an individual patient) is developed to predict the incidence of severe PJP in HIV and non-HIV patients. The nomogram could optimally assist in reducing poor outcomes and can be used for efficient estimation of prognosis of patients in PJP infections. 95

## Future Directions

Tables 4 and 5 describe ongoing clinical trials of PJP infection. There are three drug trials (TMP-SMX, steroids, and caspofungin) in non-HIV patients that will assess the mortality within 28 days and response to treatment with study drugs. The next two drug trials are investigating the duration of steroids and dosing strategy of TMP-SMX for the PJP infections in the immunocompromised patients (including HIV and non-HIV patients). The rest of them are focused on new trails primarily targeting diagnostic tools.

**Table 4:** Ongoing trials involving different aspects of PJP. Not applicable (N/A) is used to describe trials without FDA-defined phases, including trials of devices or behavioral interventions

Drug/treatment under trial	NCT	Randomized clinical trial assignment	Route	Interventions	Description/end point/outcome
Sulfanilamides, caspofungin and corticosteroids (PNEUMOQUANT)	NCT02603575	Cross over	Oral (sulfanilamides) IV (caspofungin and corticosteroids)	on the base of sulfanilamides with caspofungin and without caspofungin group and corticosteroids and without corticosteroids	Safety and efficacy of caspofungin and corticosteroids in non-HIV patients with *Pneumocystis pneumonia*
Methylprednisolone	NCT02944045	Parallel	IV	Methylprednisolone vs. placebo	Effect of steroids during pneumocystis infection among non-HIV immunocompromised patients (PIC)
Prednisolone or equivalent (with methylprednisolone)	NCT03856229	Parallel	Oral	Conventional 21 day scheduled vs. shortened scheme	Short-term steroid in patients with moderate and severe PJP in HIV patients
TMP-SMX	NCT04851015	Parallel	Oral or IV	Reduced-dose TMP-SMX (10mg/kg/day) vs. standard dose (15 mg/kg/day)	Low-dose TMP-SMX for the treatment of PJP

Abbreviations: FDA, Food and Drug Administration; HIV, human immunodeficiency virus; IV, intravenous; PJP, *Pneumocystis jirovecii* pneumonia; TMP-SMX. trimethoprim-sulfamethoxazole.

**Table 5:** Ongoing trials on diagnostic tools of PJP

Diagnostic test under trial	NCT	Trial phase	Intervention model	Interventions	Description	Primary endpoint
PCR on oropharyngeal rinse	NCT02648256	N/A	Randomized single-group assignment	Fungal quantification via PCR in bronchoalveolar lavage vs. oropharyngeal rinse	Evaluation of PCR on oropharyngeal rinse and establish fungal threshold(copies/ml) for Bronchoalveolar lavage.	Precision rate of PCR test on oropharyngeal sample
Noninvasive diagnosis of pneumocystis pneumonia (DANIPOP)	NCT03613025	N/A	Randomized parallel assignment	Respiratory samples (oral secretions, sputum, and bronchial aspirates) vs. bronchoalveolar lavage, β-D-glucans testing on serum and radioclinical evidence	Diagnostic value of nontargeted and/or noninvasive respiratory samples with real time PCR for the rapid diagnosis of Pneumocystis pneumonia	Sensitivity, specificity, NPV, PPV
SPM in *Pneumocystis jirovecii* pneumonia (INFLA-PCP)	NCT03606252	N/A	Randomized parallel assignment	SPM (lipoxins, maresins, protectins and resolvins, etc.) levels for evaluation in Pneumocystis pneumonia human infection	High levels of SPM vs. low levels of SPM could be predictive evolution of the disease, in adequate pneumocystis therapy conditions	14,15-DHET blood level at the inclusion, 14,15-DHET blood level at day 7
New techniques for using a saline wash as a diagnostic tool for *Pneumocystis* pneumonia	NCT00342589	N/A	Case–control	Collection of respiratory secretion specimens and blood samples, identification of genome and its variation associated with drug resistance. epidemiology of pneumocystis by comparison with health volunteers	Detection of PJP using nuclear amplification tests in saline oral wash	Collection of respiratory samples for laboratory evaluation
Pneumocystis jirovecii DNA extraction and analysis in EBC	NCT04358419	N/A	Case–control	Detection of *Aspergillus* specific proteins and *Pneumocystis jirovecii* DNA in EBC	Protein and DNA extraction analysis of EBC in pulmonary Aspergillosis and PJP *,* respectively	Identification of biomarkers in the EBC from pulmonary Aspergillosis patients with proven/probable/possible infection. Secondary outcome (substudy) detection of pneumocystis DNA particles in the EBC

Abbreviations: 14,15-DHET, 14,15-dihydroxyeicosatrienoic acid; EBC, exhaled breath condensate; N/A, not available; NPV, negative predictive value; PCR, polymerase chain reaction; PJP, *Pneumocystis jirovecii* pneumonia; PPV, positive predictive value; SPM, specialized proresolving mediators.

## Conclusion

PJP is a serious infection primarily of the lungs that can result in death if not treated in timely fashion. Even the incidence of PJP in HIV infection has decreased, a greater percentage of patients with PJP infection is comprised of individuals with non-HIV immunocompromising conditions. HIV patients have mild-to-severe symptoms, while non-HIV patients present with a rapidly progressing disease. TMP/SMX is first-line drug for treatment and has proven to be highly effective for PJP prophylaxis in both HIV and non-HIV patients. While several diagnostic tests with treatment and prophylactic regimes are available at our disposal, there is need for more research to prevent and manage this disease more effectively.
